# The therapeutic targets of N6-methyladenosine (m6A) modifications on tumor radioresistance

**DOI:** 10.1007/s12672-023-00759-3

**Published:** 2023-07-31

**Authors:** Yi Zhang, Wendong Gu, Yingjie Shao

**Affiliations:** Department of Radiation Oncology, The Third Affiliated Hospital of Soochow University, 185 Juqian Street, Changzhou, 213003 China

**Keywords:** Tumor, m6A, Radiotherapy, Radioresistance

## Abstract

Radiation therapy is an important tool for malignant tumors, and its tolerance needs to be addressed. In recent years, several studies have shown that regulators of aberrant m6A methylation play an important role in the formation, development and invasion and metastasis of tumors. A large number of studies have confirmed aberrant m6A methylation as a new target for tumour therapy, but research on whether it can play a role in tumor sensitivity to radiotherapy has not been extensive and thorough enough. Recent studies have shown that all three major enzymes of m6A methylation have significant roles in radioresistance, and that the enzymes that play a role differ in different tumor types and by different mechanisms, including regulating tumor cell stemness, affecting DNA damage and repair, and controlling the cell cycle. Therefore, elucidating the mechanisms of m6A methylation in the radiotherapy of malignant tumors is essential to counteract radioresistance, improve the efficacy of radiotherapy, and even propose targeted treatment plans for specific tumors. The latest research progress on m6A methylation and radioresistance is reviewed in this article.

## Introduction

The occurrence and development of tumors globally have accelerated rapidly in recent decades. Malignant tumors have severely threatened the health and lives of people. According to the information released by the International Agency for Research on Cancer (IARC) under the World Health Organization (WHO), the number of patients with malignant tumors exceeded 19 million in 2020, while the number of deaths was nearly 10 million worldwide. Studies estimate a 50% increase in the global cancer burden by 2040 [1, 2]. Malignant tumors are the leading cause of mortality among various diseases, with a low average survival time. Therefore, there is an urgent need to develop an effective anti-tumor treatment strategy, with radiotherapy being a critical component of such treatment.

Radiation therapy causes DNA fragmentation by irradiating the target area with concentrated rays to kill tumor cells [3, 4]. This treatment modality is highly accurate, atraumatic, and rapid. Therefore, it could be used either as a radical treatment alone or in combination with chemotherapy, immunotherapy, targeted therapy, and other enhanced therapeutic regimes, as well as neoadjuvant or adjuvant regimens when performed preoperatively or after surgery. This approach is applicable to several tumors in different bodily systems [5–8]. However, similar to most treatment schemes, tumors can also develop resistance to radiotherapy, potentially due to factors such as the level of DNA damage repair, stemness of tumor cells, rapid proliferation, metastasis, invasion, and other pathways. There is no in-depth study on how to slow down radiation resistance [9–14].

**Fig. 1:**
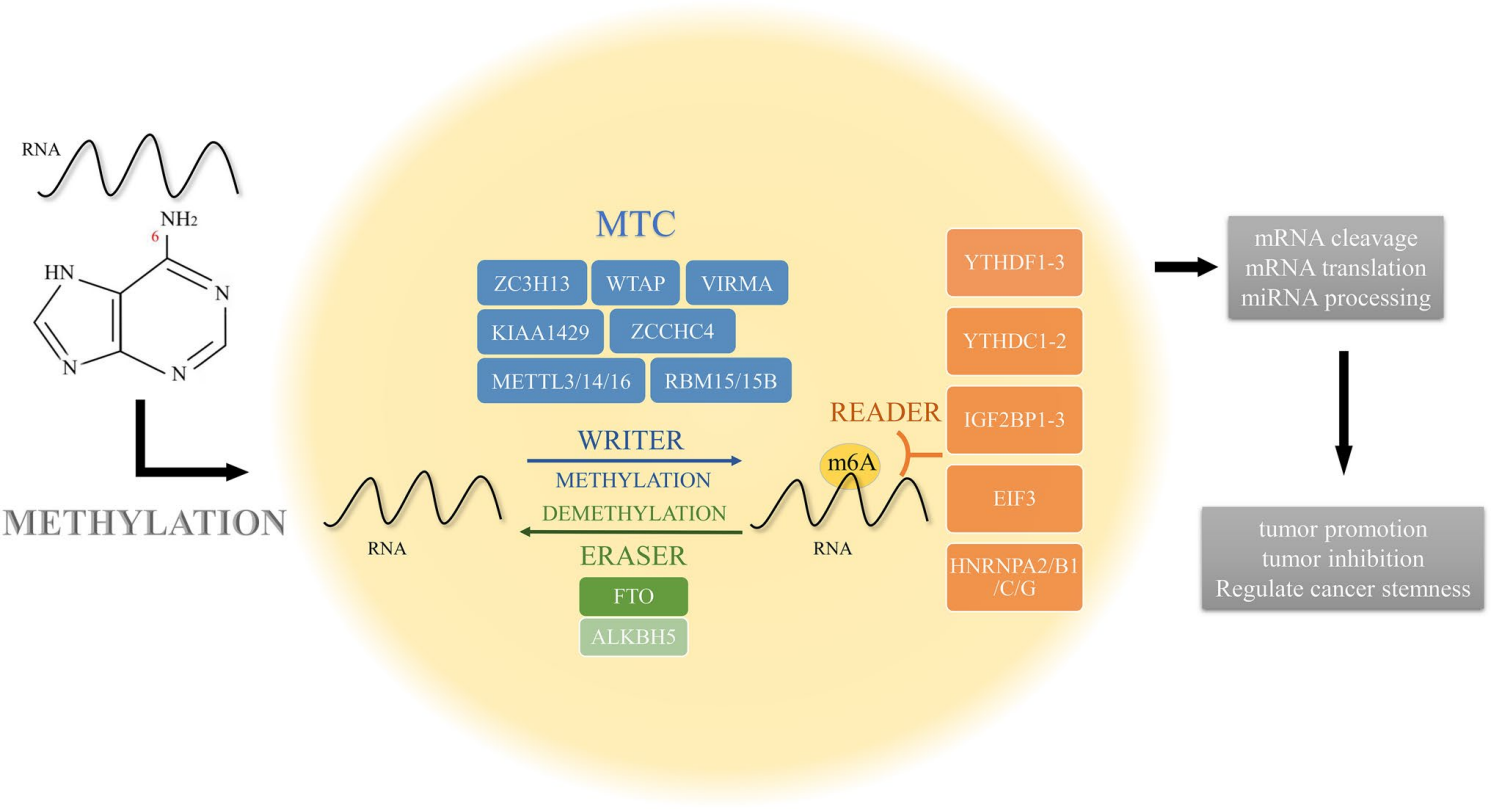
The regulatory mechanism of m6A methylation. RNA's 6-methyladenine (N6) is subjected to a methylation modification process known as "m6A methylation," which involves three key enzymes: "writer," "eraser," and "reader." The "writer," or m6A methylation complex (MTC), is one of them and mostly consists of METTL3/5/14/16, WTAP, and other proteins. FTO and ALKBH5 make up the majority of the eraser. The YTH family, IGF2BP1-3, eIF3, HNRNPC/G, and HNRNPA2/B1 are the primary readers. Numerous mRNA-related processes, such as mRNA splicing, translation, and miRNA processing, include m6A methylation. It contributes to the development of tumor stemness and either promotes or inhibits tumor growth

M6A methylation widely exists among various RNA modifications, including methylation, deamination, thiolation, and acetylation (Fig. 1) [15–20]. M6A methylation occurs on 6-methyladenine (N6) of RNA under the action of RNA methyltransferase complex (MTC). This selective addition of methyl groups to the specific adenine bases is a reversible process. The process of m6A methylation includes methylation, demethylation, and m6A methylation recognition, where m6A can be added or removed. Three major enzymes are involved in the process: m6A methylases, demethylases, and methylation recognition proteins [21–28]. MTC mainly includes METTL3, METTL14 and WTAP, of which METTL3 and METTL14 form stable complexes in a 1:1 ratio [23]. The former mainly plays a catalytic role, while the latter recognizes specific RNA sequences as catalytic substrates and stabilizes the MTC structure. WTAP is not related to catalysis and mainly recruits METTL3/6 to facilitate m14A installation [24, 29–32]. RNA-binding motif protein 15/15B (RBM15/15B) is an additional component of the MTC and interacts with METTL3 in a WTAP-dependent manner [33]. VIRMA, KIAA1429, is a vir-like m6A methyltransferase that acts primarily as a preemptive recruiter of MTC-mediated methylation of adenine bases near the 3′ UTR [34]. ZCCHC4 mainly methylates human 28S rRNA [35]. ZC3H13 is a zinc finger protein that is involved in the regulation of RNA and anchoring WTAP in the nucleus [36]. METTL16 is an independent m6A methyltransferase, which mainly acts as a shear regulator [37, 38]. The m6A demethylases are enzymes that demethylate adenosine that has undergone m6A methylation and mainly include the fat obesity-associated protein (FTO) and the ALKB homolog (ALKBH5/3) [39, 40]. FTO catalyzes the demethylation of 3-methylthymine in single-stranded DNA by iron(II) and 2-oxoglutarate-dependent oxygenases [39]. ALKBH3 efficiently demethylates 1-methyladenine (1-meA) and 3-methylcytosine (3-meC) in endogenously methylated RNA [41]. ALKBH5 reverses m6A in mRNA by oxidation in vitro and in vivo [40]. Methylation recognition proteins, which recognize adenosine that has undergone m6A methylation, recruit a variety of binding proteins to perform or regulate various functions and activities, either directly or indirectly. The YTH family, which contains the YTH structural domain, is the main methylation recognition protein, including YTHDC1/2 and YTHDF1/2/3. The m6A methylation recognition proteins also include the conserved single-stranded RNA binding proteins (RBPs) IGF2BPs (IGF2BP1/2/3), eukaryotic initiation factor 3 (eIF3), and the HNRNP family (HNRNPA2/B1, HNRNPC/G) [42–46]. Different readers have different m6A positioning functions. Nuclear M6A readers include YTHDC1, HNRNPA2B1, HNRNPC11 and HNRNPG [45, 47, 48]. Cytoplasmic m6A readers contain YTHDF1/2/3, YTHDC2 and IGF2BP1/2/3 [42, 49, 50]. M6A mainly exists in the promoter region, stop codon region and RRACH motif of mRNA, and is involved in various mRNA-related activities, including mRNA splicing, translation, and miRNA processing [47, 51–55].

**Fig. 2:**
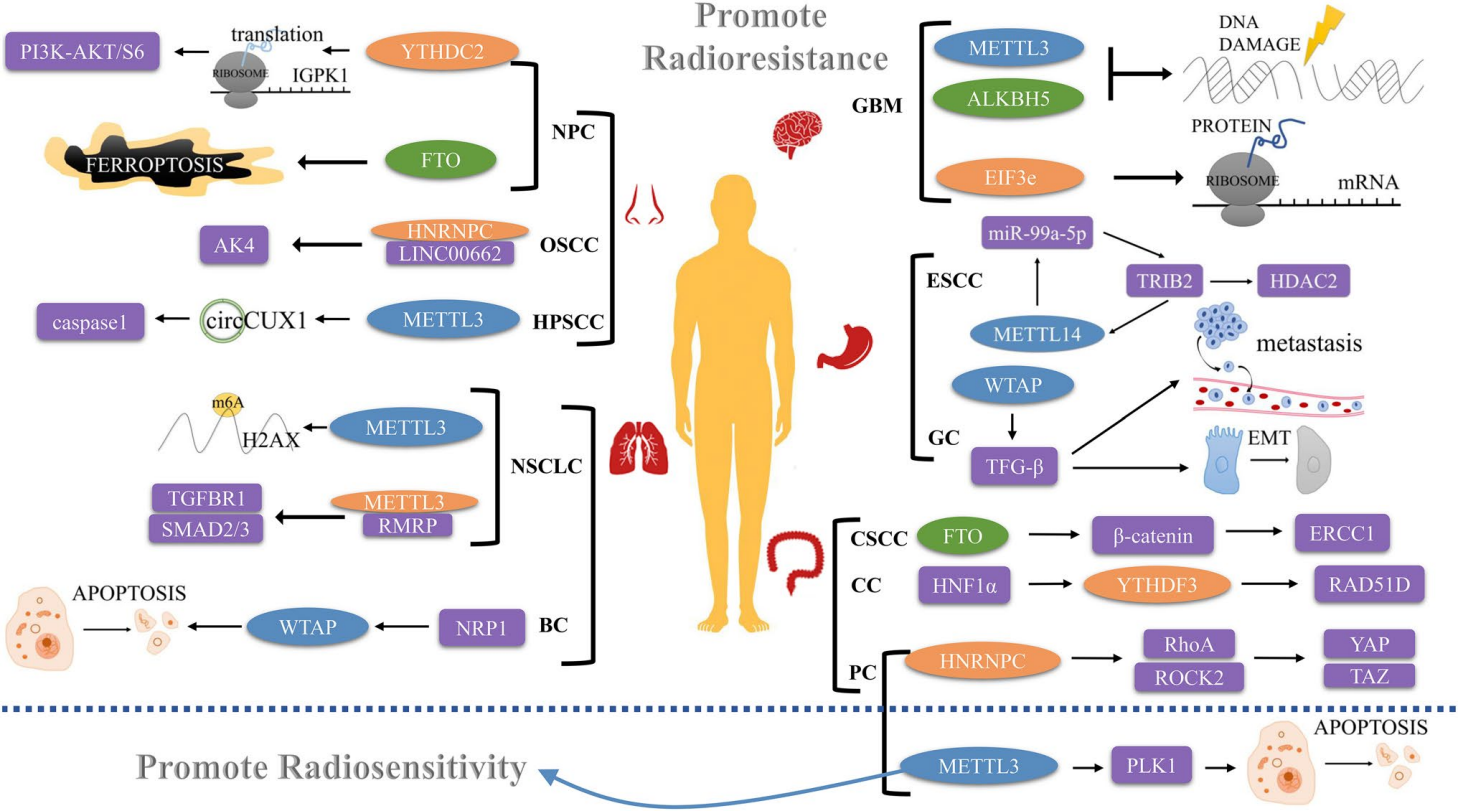
Mechanism of action by which m6A methylation affects tumor radiosensitivity. The m6A methylation process can regulate the expression of related molecules or activate certain pathways, which in turn affects the radiosensitivity of tumors by regulating DNA damage and repair, apoptosis, and distant metastasis

The relationship between m6A methylation and cancer occurrence and development has been studied extensively. While reviews have focused on tumor chemotherapy resistance, there is a lack of systemic reviews on the impact of m6A methylation on radiotherapy. This review aims to provide a comprehensive summary of the current understanding of the role of m6A methylation-related genes in tumor radiotherapy (Table 1, Fig. 2). We further aimed to identify potential targets for radiosensitization from the perspective of m6A methylation.

**Table 1:** The role of m6A methylation modulators in regulating tumor radiosensitivity

Cancer types	M6A methylase	Type	Target factor	Mechanism	References
GBM	METTL3	Writer	SOX2	METTL3 enhances DDR through SOX2 dependence, and METTL3 silencing in GBM enhances radioresistance	[58]
GBM	ALKBH5	Eraser	GBMSCs	The downregulation of ALKBH5 limits DDR, thereby promoting radioresistance	[60]
GBM	eIF3e	Reader	HIF, ALDH1A	The upregulation of eIF3e affects the mRNA translation of the relevant gene, leading to radioresistance	[67]
NPC	FTO	Eraser	OTUB1	FTO promotes radioresistance by inhibiting radiation-induced cellular iron death in an OTUB1-dependent manner	[68]
NPC	YTHDC2	Reader	IGF1R	Upregulation of YTHDC2 expression can promote the mRNA translation of IGFR1, leading to the activation of the PI3K-AKT/S6 pathway, and ultimately promoting radioresistance	[69]
OSCC	HNRNPC	Reader	LINC00662/HNRNPC/AK4	LINC00662 upregulates AK4 expression by binding to HNRNPC, resulting in radioresistance	[71]
HPSCC	METTL3	Writer	Caspase1	METTL3 stabilizes circCUX1 expression, and circCUX1 can downregulate caspase1 expression, thereby promoting radioresistance	[73]
ESCC	METLL14	Writer	miR-99a-5p/TRIB2	METTL14/miR-99a-5p/TRIB2 positive feedback activates the TRIB2/HDAC2 axis, ultimately resulting in radioresistance	[77]
BC	WTAP	Writer	WTAP/NRP1	NRP1 confers stemness on BC cells and inhibits radiation-induced apoptosis through WTAP, thereby promoting radioresistance	[81]
NSCLC	METTL3	Writer	H2AX	METTL3 regulates the m6A modification of H2AXmRNA to regulate the expression of H2AX, thereby promoting radioresistance	[83]
NSCLC	METTL3	Writer	TGFBR1/SMAD2/SMAD3	RMRP regulates the TGFBR1/SMAD2/SMAD3 pathway, and METTL3 modifies RMRP and enhances its stability, ultimately leading to radioresistance	[82]
GC	WTAP	Writer	TFG-β	WTAP promotes GC transfer and EMT formation by upregulating TFG-β, which in turn leads to radioresistance	[87]
PC	METTL3	Writer	PLK1	METTL3 regulates PLK1 in a cell cycle-dependent manner, and PLK1 3'UTR demethylation disrupts cellular homeostasis, promotes apoptosis, and enhances radiosensitivity	[88, 89]
PC	HNRNPC	Writer	RhoA/ROCK2-YAP/TAZ	HNRNPC regulates the RhoA/ROCK2-YAP/TAZ axis to promote radioresistance	[91]
CSCC	FTO	Eraser	β-catenin, ERCC1	FTO upregulates β-catenin expression, and ERCC1 is involved, ultimately leading to radioresistance	[96]
CC	YTHDF3	Reader	HNF1α/YTHDF3/RAD51D	Promotes radioresistance via the HNF1α/YTHDF3/RAD51D axis	[97]

GBM: glioblastoma multiforme; HPSCC: hypopharyngeal squamous cell carcinoma; NPC: nasopharyngeal carcinoma; OSCC: oral squamous cell carcinoma; BC: breast cancer; ESCC: esophageal squamous cell carcinoma; LUAD: lung adenocarcinoma; NSCLC: non-small cell lung cancer; CC: cervical cancer; CSCC: cervical squamous cell carcinoma; PC: Pancreatic cancer; GC: gastric cancer

## Effect of m6A methylation on radioresistance of tumors

### Glioblastoma

Glioblastoma multiforme (GBM), a high-grade brain glioma with a poor prognosis, has a median survival time of about 1 year after diagnosis [56]. Despite the potential benefits of radiotherapy or chemoradiotherapy as a postoperative or biopsy treatment, radiotherapy resistance remains a persistent challenge [57].

#### METTL3

Studies show that the overall m6A modification and METTL3 level in GBM are high compared with differentiated glioma cells (DGCs). METTL3 downregulation has been shown to reduce the expression of stem cell-specific markers SSEA1, glioma reprogramming factors POU3F2, SOX2, SALL2, OLIG2, neurosphere formation, and the proportion of viable cells. On the other hand, the number of apoptotic cells increases [58]. Studies have shown that METTL3 is highly expressed in GBM and plays an important role in its formation, maintenance, and recurrence.

The radiation resistance of GBM caused by METTL3 was confirmed and further explored. Visvanathan et al. found that METTL3 modifies SOX2 through m6A modification, enhancing the stability of m6A modification of SOX2 mRNA. Additionally, in vivo m6A modification and METTL3 binding required three METTL3 and SOX2 sites on SOX2 3’UTR. Silencing METTL3 in brain glioma stem cells was observed to increase their sensitivity to γ-rays and block DNA repair, as demonstrated by the accumulation of γ-H2AX. Moreover, METTL3 silencing was found to rescue the formation of neurospheres, which further enhances the radiosensitivity of brain gliomas [58, 59]. These findings indicate that METTL3 is a powerful potential target for overcoming radiation resistance in brain gliomas.

#### ALKBH5

Kowalski-Chauve et al. found that the downregulation of ALKBH5 mRNA level increases the radiosensitivity of GBMSCs [60]. Further analysis of related genes showed an upregulation after irradiation in the phosphorylation of CHK1, the expression of HHR, NHEJ-related genes, and the expression of histone H2AX, a marker of DNA damage (γ-H2AX), all of which returned to normal levels within 24 h. DNA repair was shown to occur after irradiation, while ALKBH5 knockdown by siRNA transfection significantly reduced the expression of HHR-related genes.γ- H2AX continued to rise within 24 h, and the increase induced by radiation was also inhibited; however, no significant change was observed in NHEJ [60]. These results suggest that ALKBH5 knockout could inhibit DNA repair and sensitize GBMSCs to radiation therapy.

Moreover, studies have highlighted the crucial role of recombinant enzymes, such as RAD51 and FOXM1, in the radioresistance of GBM [13, 61–66]. Kowalski-Chauve et al. found an increase in RAD51 and FOXM1 levels after irradiation, which was inhibited by ALKBH5 knockdown [60]. This partially confirmed the antagonistic effects of ALKBH5 downregulation on radioresistance. The study further demonstrated that the knockdown of ALKBH5 in GBMSCs reduced the expression of genes involved in GBM radioresistance and could inhibit the ability to repair DNA, leading to increased sensitivity to radiation therapy. Thus, ALKBH5 could be a promising target for enhancing the radiosensitivity of GBM.

#### eIF3e

EIF3e is an important reader protein of m6A. Bertorello et al. found enhanced eIF3e mRNA and protein expression levels in GBM cells and their association with tumor grade, i.e., high expression in high-grade gliomas and low expression in low-grade gliomas. An additional survey study of gliomas that recurred after chemoradiotherapy found an increase in the proportion of tumor cells positive for eIF3e with an upregulated protein expression in the recurrent tumors. Regions of eIF3e expression in brain glia were also characterized, demonstrating an upregulation in tumor microvessels and pseudopuncta [67]. Further studies illustrated that eIF3e could regulate the expression of HIF and the GSC marker ALDH1A; downregulation of eIF3e decreases the protein level of HIF, which could be partially reversed by hypoxia [67]. Moreover, EIF3e could selectively affect the translation of related mRNAs. For example, the knockdown of eIF3E increases the translation of P53-related DADD45α and FAS, while reducing the translation of UBE2V1 and CDC45 related to cell survival, DNA replication, and repair; it also had the opposite effect on the protein expression levels. This result has been validated in many different GBM cell lines. Further studies show that eIF3e impacts the selective translation of mRNA through the unique combination of eIF3e, eIF3d, and DDX3X with co-target mRNA [67]. Radiation treatment in eIF3e depleted GBM cell line (U251), and radiation-resistant GBM cell line (LN18) exhibited a decreased cell survival rate in both cell lines [67]. These results suggest that eIF3E could affect the radioresistance of GBM, and inhibiting eIF3E could enhance its sensitivity to radiation.

### Nasopharyngeal carcinoma

Nasopharyngeal carcinoma (NPC) is a malignant tumor of the nasopharynx. It affects all age groups and has a high incidence in southern provinces of China. Nasopharyngeal carcinoma is highly sensitive to radiotherapy, making it crucial to resolve the radiation resistance of nasopharyngeal carcinoma.

#### Fat mass and obesity-associated protein (FTO)

Studies have suggested that ferroptosis plays a significant role in mediating cell death through radiation therapy. Several studies have reported that ferroptosis results in a characteristic mitochondrial morphology during ferroptosis, an increase in lipid peroxides, and a decrease in glutathione (GSH) levels. These findings indicate iron-induced cell death occurs after radiotherapy, and using iron death inducer erastin can enhance tumor radiosensitivity [68].

Radiation resistant cell lines were generated by irradiating C666-1 and HONE1 cell lines of nasopharyngeal carcinoma, and a comparison of their respective FTO expression levels revealed that FTO was expressed at higher levels in the radiation resistant cell lines, i.e., FTO indeed had a close relationship with the cell radiation resistance of nasopharyngeal carcinoma. After irradiation of cells with high expression of FTO, it was found that the DNA damage was lower compared to the cells with normal expression, while the DNA damage of cells with FTO inhibitor FB23-2 was aggravated after irradiation, suggesting that FTO could reduce radiation-induced DNA damage and induce resistance to radiation in NPC cells [68].

Huang et al. further found that the expression of GSH increased and the expression of lipid peroxide decreased upon FTO overexpression; however, after the inhibition of FTO by FB23-2, NPC cells exhibited mitochondrial atrophy, decreased GSH expression and increased lipid peroxide expression. The use of iron death inhibitor FER-1 could save the cell death caused by FB23-2. These results indicate that FTO inhibits iron death, leading to an increase in the radioresistance of NPC cells [68].

Through database analysis, Huang et al. further identified OTUB1 as a potential downstream target of FTO. Subsequent studies confirmed that OTUB1 levels were enhanced in anti-radiation cells, and its protein and mRNA expression levels increased with the increase in FTO expression. OTUB1 has been shown to participate in iron death by stabilizing SLC7A11. This study showed that FTO overexpression could stabilize the interaction between OTUB1 and SLC7A11, indicating that FTO inhibited iron death through the OTUB1/SLC7A11 pathway [68].

To sum up, FTO plays an important and positive role in the generation of radiation resistance of NPC and could inhibit iron death through the OTUB1/SLC7A11 pathway. FTO inhibitor FB23-2 and iron death inducer erastin could effectively enhance radiosensitivity, and therefore, these modulators could be potential targets for antagonizing the radioresistance of NPC.

#### YTHDC2

He et al. conducted a study on the effect of m6A-related protein on the radiotherapy of NPC and found that the mRNA and protein expression of the m6A reader YTHDC2 was regulated in radio-resistant NPC cells. Knockdown of YTHDC2 decreased cell survival rate, slowed down colony growth, decreased cell proliferation, and increased apoptosis after irradiation. Consistently, overexpression of YTHDC2 showed the opposite results [69]. The study further revealed that the PI3K-AKT/S6 pathway was significantly activated in radiation-resistant NPC cells and that the expression of YTHDC2 was positively correlated with the translation efficiency of insulin-like growth factor-1 receptor (IGF1R). The follow-up study confirmed that high expression of YTHDC2 in NPC cells promoted the translation efficiency of IGF1R mRNA, thus activating the PI3K-AKT/S6 signal pathway, resulting in the increased anti-radiation ability of cancer cells [69]. Hence, these findings suggest that the high expression of YTHDC2 could promote radiation resistance and be a potential target for increasing the sensitivity of NPC to radiotherapy.

### Oral squamous cell carcinoma

Oral squamous cell carcinoma (OSCC) is a highly invasive head and neck tumor, with radiotherapy being its effective treatment. However, OSCC cells have strong radiation resistance, making it imperative to find effective therapeutic targets and enhance radiosensitivity [70]. Chen et al. found that the oncogene LINC00662 has a close relationship with the radiosensitivity of OSCC, and knocking out LINC00662 could effectively enhance radiosensitivity. As an m6A recognition protein, HNRNPC is closely related to tumor initiation and development. Further research found that LINC00662 increases the expression of AK4 by binding to HNRNPC protein and enhances the radiation resistance of OSCC through AK4 overexpression. Subsequent experiments confirmed that AK4 overexpression could rescue the enhanced radiosensitivity of LINC00662 [71]. To sum up, LINC00662 plays an important role in the radioresistance of OSCC cells by upregulating the expression of AK4 in combination with HNRNPC and could potentially become a potent target for enhancing the efficacy of radiotherapy on OSCC.

### Hypopharyngeal squamous cell carcinoma

Hypopharyngeal squamous cell carcinoma (HPSCC) is one of the most common malignant tumors in otolaryngology and head and neck surgery with the worst prognosis. Patients with early HNSCC could achieve good results through surgery or radiotherapy, but the prognosis of late patients is often not ideal [72]. Wu et al. conducted in-depth research on the expression and function of m6A-regulated circRNA in HNSCC. They found that the expression of circCUX1 was upregulated in patients with HPSCC with radiation resistance and poor prognosis. CircCUX1 knockdown promoted the sensitivity of HPSCC cells to radiation, suggesting that circCUX1 would cause radiation resistance in hypopharyngeal cancer [73]. Studies have shown that Caspase-1 could directly cut IL-1β precursor, and IL-18 precursor and release them to the extracellular region, inducing an inflammatory response and participating in the progression of inflammation-related tumors [74]. In addition, CircCUX1 could bind to Caspase1 and inhibit its expression, resulting in reduced release of inflammatory factors. These findings indicated that the radioresistance of hypopharyngeal cancer cells promoted by CircCUX1 depends on the Caspase-1 pathway. Meanwhile, the study also found that METTL3 mediated the m6A methylation and stable expression of circCUX1 [73]. Therefore, circCUX1 and METTL3 may serve as potential therapeutic targets for enhancing radiosensitivity in HPSCC.

### Esophageal carcinoma

Esophageal squamous cell carcinoma (ESCC) is a highly invasive and treatment-resistant tumor [75, 76]. Liu et al. studied the effect of m6A methylation in ESCC on radiation resistance. They found a key role of the METTL14/miR-99a-5p/TRIB2 axis and proposed its correlation with ESCC radiation resistance. The decrease in METTL14 activity downregulates the expression of miR-99a-5p in ESCC CSCs and further upregulates TRIB2, which further inhibits the expression of METTL14 through protein hydrolysis mediated by COP1. The inhibited expression of METTL14 continues to downregulate the expression of miR-99a-5p in ESCC CSCs. Finally, it reaches positive feedback to balance the expression of several proteins in patients with ESCC, which combinatorically produces effects. In addition to the METTL14/miR-99a-5p/TRIB2 axis, TRIB2 activates HDAC2 and inhibits HDAC2 (Ser394) phosphorylation through the Akt/mTOR/S6K1 signal pathway in ESCC, thus inhibiting cancer stemness and improving radiation resistance of ESCC cells [77]. To sum up, the discovery of METTL14/miR-99a-5p/TRIB2 axis and TRIB2/HDAC2 axis provides a target for radiosensitization of ESCC.

### Breast cancer

Breast cancer (BC) is a common malignant tumor in women, with radiotherapy being a necessary treatment [78–80]. Wang et al. found that NRP1 overexpression led to an upregulation in the protein and mRNA expression levels of BC cell stem cell markers such as Nanog and Oct4, indicating that NRP1 was closely related to BC cell stemness. Wang et al. further studied the association between NRP1 and radiation resistance and showed that the combination of highly expressed NRP1 treatment significantly reduced double-stranded DNA (dsDNA) breaks compared with radiation alone [81]. These findings indicated that NRP1 played an active role in the generation of BC radiation resistance. Further studies showed that WTAP expression decreased in a dose-dependent manner after irradiation. WTAP downregulation suppressed apoptosis caused by NRP1 overexpression, while NRP1 knockout downregulated the expression of WTAP and bcl-2. Radiotherapy, in conjunction with the knockout of NRP1, resulted in a more pronounced reduction in tumor size and a greater decrease in the expression of WTAP and bcl-2 compared to radiotherapy alone [81].

### Non-small cell lung cancer

Non-small cell lung cancer (NSCLC) is one of the most common malignant tumors in the lung. Most patients are in the middle and advanced stages when they are diagnosed. Depending on the patient’s situation, the treatment methods could include surgical treatment, drug treatment, and radiotherapy. However, each treatment method has its advantages and limitations. Among them, resistance generated in radiotherapy leads to poor efficacy and prognosis. Therefore, it is urgent to find new targets for radiation resistance.

#### METTL3

Yin et al. found that RMRP was highly expressed in NSCLC, involved in tumor progression, and associated with a low survival rate. According to the findings of several follow-up studies, RMRP is related to the expression of transcription factor YBX1 and promotes the transcription of TGFBR1 by recruiting YBX1 to the TGFBR1 promoter. This recruitment, in turn, regulates the TGFBR1/SMAD2/SMAD3 pathway to promote the enhancement of CSCs, EMT, and spheroid formation of ESCLC, ultimately leading to enhanced radiation resistance. The m6A methylase METTL3 plays a role in modifying RMRP and improving its transcriptional stability in this pathway. METTL3 knockout leads to a decrease in the expression of TGFBR1, while METTL3 overexpression increases the enrichment of YBX1 at the TGFBR1 promoter and the bindings of YBX1 and RMRP [82]. Moreover, Xu et al. found that METTL3-mediated m6A methylation in NSCLC cells increased dose-dependently under carbon ion irradiation. These findings showed that the proliferation, migration, and invasion of METTL3 knockout NSCLC cells were significantly inhibited. Additionally, these cells exhibited an increased level of γH2AX, with a damaged epithelial-mesenchymal transformation (EMT) phenotype, characterized by increased E-cadherin and decreased Vimentin and Snail proteins. Consistently, METTL3 overexpression cells had the opposite expression [83]. These results suggested that METTL3 plays a vital role in the radioresistance of NSCLC.

#### IGF2BPs

Hao et al. found a dose-dependent increase in the expression of VANGL1 in irradiated NSCLC cells. VANGL1 was shown to stimulate the BRAF/TP53BP1/RAD51 cascade reaction to induce DNA repair and produce radiation resistance. They further showed that VANGL1 downregulation could significantly enhance the radiation damage of NSCLC cells. The increase in VANGL1 mRNA stability was related to the increase in m6A level, indicating that the m6A-related genes METTL3 and IGF2BP2/3 promoted the expression of VANGL1 by improving mRNA stability, thereby regulating the radiation resistance of NSCLC cells [84].

#### SETD2

Zeng et al. found that SETD2-depleted NSCLC cells exhibited decreased proliferation and metastasis, increased cell apoptosis, and reduced DNA repair, which might be related to the enhancement of the radiosensitivity of NSCLC cells. In addition, SETD2 was associated with a favorable prognosis, and its protective effect on the prognosis of NSCLC increased with the decrease of m6A reader RBM15 and YTHDF3; however, the correlation between m6A and SETD2 in radiosensitivity is still unclear and needs further research [85].

### Gastric cancer

WTAP has been shown to be highly expressed in gastric cancer and is associated with a poor prognosis [86]. Liu et al. showed that WTAP increased the propensity for metastasis and enhanced the ability of gastric cells to undergo epithelial-mesenchymal transition (EMT). WTAP also improved the expression and stability of TFG-β mRNA. WTAP knockout alleviated these manifestations but also increased the resistance of gastric cancer to multiple combined chemotherapy and radiation [87]. The current research on WTAP and the radiosensitivity of gastric cancer is lacking. Therefore, further research on WTAP is required to establish it as a target for the clinical treatment of gastric cancer.

High WTAP expression in stomach cancer is associated with a bad prognosis, according to studies [86]. Liu et al. showed that WTAP increased the likelihood that gastric cancer would spread, made the cells more susceptible to epithelial mesenchymal transformation (EMT), and enhanced the expression of TFG- and mRNA stability. These manifestations were lessened when WTAP was defeated. The study also discovered that WTAP boosted stomach cancer's resistance to radiation and multiple combination chemotherapy [87]. WTAP is anticipated to be a new target for the clinical therapy of gastric cancer. Currently, research on WTAP and gastric cancer radiosensitivity is few and inadequately detailed.

### Pancreatic cancer (PC)

Pancreatic cancer (PC) is a relatively common malignant tumor of the digestive tract. It lacks typical clinical symptoms in the early stage, so its diagnosis rate is low, with high malignancy and rapid progress. It has high drug resistance to all existing therapies, including radiotherapy, and is one of the cancers with the worst prognosis. Therefore, finding and applying therapeutic targets against PC radiation resistance is crucial.

#### METTL3

The study by Taketo et al. showed that the high expression of m6A methyltransferase METTL3 in PC cell lines was closely related to the therapeutic resistance of PC and was considered a potential therapeutic target of PC. They showed that METTL3 downregulation increased the proportion of apoptotic cells, indicating that the deletion of METTL3 could improve the radiosensitivity of PC cells. However, Taketo et al. postulated that the regulation of MAPK cascade and cellular processes by METTL3 was related to the radiochemical resistance of PC cells. Their findings indicated that the regulation of ubiquitin-dependent processes might eventually lead to genomic instability and damage to DNA repair, while the regulation of RNA splicing might lead to unexpected splicing and ultimately lead to insufficient repair of DNA damage. Therefore, METTL3 could lead to the radioresistance of PC cells; however, the target gene of METTL3 is still unexplored [88].

Tatekawa et al. further confirmed that PLK1 is one of the important genes regulated by METTL3 through RNA methylation, which was closely related to PC. The expression of PLK1 was upregulated in PC, and it was found that the increased expression of PLK1 was highly related to the poor prognosis of patients with PC. They further used a selective small molecule inhibitor of PLK1, NMS-P937, and found that its use at the same time of irradiation could activate the ATR/CHK1 pathway and induce G2/M phase arrest, increasing the number of double-stranded broken DNA (DSBs), eventually leading to increased cell apoptosis. This observation indicated that PLK1 inhibition could increase radiosensitivity. At the same time, it was found that both PLK1 and METTL3 were upregulated during mitosis, and their expression was positively correlated at each stage of the cell cycle, suggesting that METTL3 regulated the expression of PLK1 in a cell cycle-dependent manner [89]. In the G2/M phase, the expression of PLK1 is upregulated [90]. They showed that a combination of IGF2BP2 and PLK1 3′UTR was involved in stabilizing and upregulating the expression of PLK1, leading to abnormal mitosis and replication stress, subsequently activating the ATR/CHK2 pathway. On the other hand, using FTO to demethyl PLK1 3’UTR downregulates PLK1, leading to mitotic disaster and cell death, thus improving radiation resistance [89].

To sum up, METTL3 could regulate the cell cycle of PC cells through the demethylation of PLK1 3’UTR, resulting in mitotic disturbance, disruption of homeostasis, and replication stress, thus increasing cell death and ultimately increasing the radiosensitivity of PC cells. Therefore, IGF2BP2/PLK1 and FTO/PLK1 might be potential targets against radiation resistance.

#### HNRNPC

Xia et al. studied the role and mechanism of m6A recognition protein HNRNPC in PC radiation resistance from the perspective of RNA m6A methylation. In this study, Xia et al. confirmed the high expression of HNRNPC and RhoA in PC cells through various methods and established the correlation between them. They showed that the high expression of HNRNPC in different cell lines increases cell viability. In contrast, the cell viability decreased, and the expression of γ H2AX was upregulated when HNRNPC was knocked out, indicating that the abnormal expression of HNRNPC was indeed related to the radiation resistance of PC. Further studies demonstrated that overexpression of HNRNPC increased the mRNA and protein expression level of RhoA and molecules in the ROCK/YAP/TAZ axis activated by RhoA. Downregulation of RhoA showed that the radiosensitivity of cells in both the HNRNPC overexpression group and the control group increased after different doses of radiation [91]. Also, fibrotic marker expression was enhanced in PC cells with increased HNRNPC expression. Knockout of HNRNPC downregulated the expression of RhoA, ROCK-2, and YAP in the cells, while the knockout of RhoA inhibited the expression of CAF-related proteins α-SAM and FAP. These findings indicate that the inhibition of HNRNPC or RhoA could restrict the activity of CAFs, thereby weakening the radiation resistance of PC [91].

### Cervical cancer

Cervical cancer (CC) has a high incidence rate and mortality among females. Its histological types are mainly divided into squamous cell carcinoma and adenocarcinoma. Although radiotherapy is an effective treatment for CC, more than 25% of patients show local or distant recurrence due to radiation resistance [92–94]. Therefore, finding new targets against CC radiation resistance is essential.

#### FTO

Li et al. confirmed the carcinogenic effect of FTO through mRNA demethylation for the first time. Zhou et al. further studied the carcinogenic effect of FTO in cervical squamous cell carcinoma (CSCC) and its relationship with radiochemotherapy resistance [95, 96].

Comparing data from various databases showed an upregulation in the expression of FTO in high-level cervical squamous intraepithelial neoplasia, CC, or poorly differentiated CSCC tissues, compared with normal cervical tissues. These findings confirmed that FTO was consistent with the invasive nature of CSCC. On this basis, Zhou et al. selected two cell lines in CSCC (siHA and C-33a) for FTO overexpression treatment and then exposed them to 2 Gy + radiation and found that their cell survival rate was higher than that of the control group, indicating that CSCC produces radiation resistance under the high expression of FTO. Furthermore, siHA increased its sensitivity to radiation upon treatment of FTO inhibitor MA2. Further research found that FTO as m6A demethylase reduced the m6A level of β-Catenin; thus, the level of expression of mRNA and protein was increased. Further research showed that ERCC1 downregulation decreased radiation resistance upon β-catenin knockdown [96].

#### YTHDF3

Du et al. found that the expression level of HNF1α mRNA and protein in CC tissue was higher, while the expression level of HNF1α mRNA in anti-radiation CC tissue was higher than the normal cervical tissue. Further studies showed that CC cell lines with different HNF1α expression levels had higher cell survival rate after irradiation, and that HNF1α overexpression was closely related to radiation resistance after forced expression or knockout of HNF1α in CC cell lines. After treatment, it was found that the over-expression of HNF1α was closely related to radiation resistance [97]. Further studies illustrated that YTHDF3 had no significant effect on the mRNA expression and stability of RAD51D but could accelerate its mRNA translation speed, thus regulating the radiation resistance of CC tissues. Subsequent studies confirmed that HNF1α, YTHDF3, and RAD51D could reverse each other’s effect. For example, the knockdown of HNF1α and RAD51D could reverse the inhibition of YTHDF3 overexpression on radiosensitivity of CC cells, and the increase of γH2AX foci caused by HNF1α overexpression could be reversed with the depletion of YTHDF3 or RAD51D in CC cells [97]. These findings indicate that the HNF1α/YTHDF3/RAD51D axis regulates the radiation resistance of CC by affecting DSB repair.

## Prospect

The research on m6A methylation has gained significant attention in recent years, with an increasing number of studies investigating its role in tumor occurrence and development. Studies have shown that m6A methylation can both promote and inhibit tumor growth and contribute to drug resistance in various anti-tumor therapies, including chemotherapy, immunotherapy, and targeted therapy [98, 99]. Here, we reviewed the latest progress of m6A methylation related to radiation resistance in tumor radiotherapy, providing promising insights and future directions for alleviating or even reversing tumor radioresistance.

As the key to promoting cancer stemness, invasion and metastasis and enhancing cancer resistance to various therapeutic means, cancer stem cells are expected to find targets in combating radiation resistance. Glioma stem cells (GSCs) in GBM represent a critical obstacle to radiotherapy resistance. Several studies have targeted GSCs to find mechanisms of action and their relevant targets associated with radioresistance, such as ALKBH5, METTL3, and eIF3e [58, 60, 67]. NRP1 also enhances stem cell properties of BC cells and confers radioresistance [81]. Additionally, tumor fibrosis can also present a degree of an impediment to radiotherapy, such as increased fibrotic markers in radioresistant PC cells. Additionally, HNRNPC has been linked to radioresistance via its involvement in the ROCK/YAP axis and fibrogenesis, as well as the elevated expression of CAF-associated proteins [91]. Besides, the invasion and distant metastasis of tumors make it more challenging to treat the tumor with radiotherapy. Exploring targets that inhibit tumor invasion could also prevent radiation resistance to a certain extent. Studies in epithelial-mesenchymal transition (EMT) have also yielded some promising results.

Radiation therapy utilizes ionizing radiation to induce DNA damage, leading to cell death and, ultimately destruction of tumors. The generation of radiation resistance is also closely tied to DNA repair ability. Whether m6A methylation or related regulators are involved in this process is still broadly unknown and worth examining. M6A methylation of RNA can regulate UV-induced DNA damage response [100], and studies have confirmed that the related genes can endow cancer cells with radiation resistance through DNA damage [101]. Consequently, the examination of new targets impacting DNA damage or the DNA repair pathway is warranted to better understand and mitigate radiation resistance in tumors. For example, METTL3 can directly regulate the expression of H2AX mRNA, an apoptotic marker, in carbon ion radiotherapy for NSCLC, thereby enhancing their sensitivity [83]. In addition, the cell cycle is also closely related to radiosensitivity; the G2/M phase is the most sensitive, the G1 phase is less sensitive, and the S phase is the most sensitive. Currently, several drugs can halt cell progression at a specific stage. One potential strategy to enhance the efficacy of radiation therapy is to target regulators that cause cell arrest in the G2/M phase and produce more DNA double-strand breaks (DSBs) to achieve an ideal tumor-killing effect. For example, inhibition of PLK1 in PC causes a massive cell arrest at the G2/M phase, leading to increased radiosensitivity of PC cells [89].

In addition, various differentiated forms of the same tumor are shown to have several therapeutic targets. For example, YTHDF3 plays a role in all types of CC, but FTO is only found to be associated with radioresistance in cervical squamous cell carcinoma (CSCC) [96, 97]. Therefore, in-depth studies of different tumor types are necessary for further validation.

Finally, it has recently been shown that single nucleotide polymorphisms (SNPs) have a role in the biological behavior of malignant tumor development, proliferation and migration, and even in the prediction of tumor risk. SNPs can act on m6A methylation to regulate tumor cell growth and proliferation by participating in the expression of related markers. For example, SNP rs5746136 (G > A) may influence m6A to modify and regulate SOD2 expression in bladder cancer by directing the binding of HNRNPC to SOD2, which is a tumor suppressor [102]; SNP rs7495G could promote HNRNPC expression in a miRNA-mediated manner, which puts pancreatic ductal adenocarcinoma at increased risk [103]; The novel SNP rs9906944 (C > T) in IGF2BP1 was significantly associated with a reduced risk of gastric cancer at discovery stage [104]. SNPs can be applied as complementary assays thus enabling a broader and more viable role for m6A in anti-malignancy therapy.

## Conclusion

Although m6A methylation has been intensively studied in various fields, its function in radiotherapy for malignancies, especially radiosensitivity, is only beginning to be understood. In-depth studies are still required to understand various aspects, such as the association of m6A methylation modulators with radiation resistance in tumors, the link between modulators associated with DNA damage or tumor stemness and radiation resistance, the involvement of cell cycle modulators in radiation resistance, the role of m6a methylation related modulators in tumor invasion and metastasis, fibrosis, the impact of angiogenesis and so on. Studies are required to further examine the roles and advantages of m6A methylation-related genes and their clinical use to provide effective treatment options for patients with cancer.

## Data Availability

The data supporting the conclusion of this review have been included within the article.
